# Structural and Functional Changes in Supraspinatus Tendinopathy through Percutaneous Electrolysis, Percutaneous Peripheral Nerve Stimulation and Eccentric Exercise Combined Therapy: A Single-Blinded Randomized Clinical Trial

**DOI:** 10.3390/biomedicines12040771

**Published:** 2024-03-30

**Authors:** Jorge Góngora-Rodríguez, Miguel Ángel Rosety-Rodríguez, Daniel Rodríguez-Almagro, Rocío Martín-Valero, Pablo Góngora-Rodríguez, Manuel Rodríguez-Huguet

**Affiliations:** 1 Department of Nursing and Physiotherapy, University of Cádiz, 11009 Cádiz, Spain; jorge.gongora@uca.es (J.G.-R.); manuel.rodriguez@uca.es (M.R.-H.); 2 Move-It Research Group, Biomedical Research and Innovation Institute of Cadiz, Puerta del Mar University Hospital, University of Cádiz, Plaza Fragela, s/n, 11003 Cadiz, Spain; 3 Department of Nursing, Physiotherapy and Medicine, University of Almería, 04120 Almería, Spain; dra243@ual.es; 4 Department of Physiotherapy, Faculty of Health Sciences, University of Málaga, 29071 Málaga, Spain; rovalemas@uma.es; 5 Doctoral School, University of Cádiz, 11003 Cádiz, Spain; pablo.gongorarodriguez@alum.uca.es

**Keywords:** percutaneous electrolysis, peripheral nerve stimulation, physical therapy, shoulder pain, supraspinatus tendinopathy, ultrasonography

## Abstract

Shoulder tendinopathies produce pain and reduce functionality. The aim of this randomized clinical trial was to analyze the effects of Percutaneous electrolysis (PE), Percutaneous peripheral Nerve Stimulation (PNS) and eccentric exercise (EE) on pain (NPRS), strength, electromyographic activity, ultrasound characteristics of the tendon (echogenicity, thickness and hypervascularization) and functionality (DASH and SPADI) in individuals with supraspinatus tendinopathy. Participants (*n* = 50) were divided into two groups; they received 4 treatment sessions, 1 per week, of PE and PNS (*n* = 25) or 10 treatment sessions of TENS and US (*n* = 25). Both groups performed the EE program consisting of 3 sets of 10 repetitions of each of the 3 exercises, twice a day, during the 4 weeks. Follow-up was carried out at 4, 12 and 24 weeks after the start of the intervention. There are statistically significant differences in the analysis between groups (*p* < 0.001) in the post-treatment and follow-up measurements favorable to the PE+PNS+EE treatment on pain (NPRS), strength, supraspinatus electromyographic amplitude, ultrasound characteristics of the tendon (echogenicity, thickness and hypervascularization) and DASH and SPADI questionnaires. The combined treatment with PE, PNS and EE is an effective option in the clinical management of tendinopathies, with positive results in the short and long term on the variables studied.

## 1. Introduction

Tendinopathies are pathological situations of the tendon characterized by a degradation process of collagen fibers [1,2,3]. The disease causes pain, reduced range of motion, and decreased function and exercise tolerance [4]. Tendon injuries stand out in the shoulder [5,6,7], especially in the rotator cuff [8,9,10], with a prevalence of 4.5% in men and 6.1% in women [11] and high socioeconomic costs.

Injury to the supraspinatus affects joint stability, especially abduction and rotation movements. This greatly impairs the ability to carry out daily living activities, for example, combing one’s hair or reaching for objects located on high shelves [9,12].

It is necessary to search for new treatment methodologies in the face of dissatisfaction with traditional medical and physiotherapeutic care protocols (rest, non-steroidal anti-inflammatory drugs and passives therapies) and the lack of consensus between them [7].

Nowadays, there are a few studies that support the effectiveness of invasive techniques such as Percutaneous Electrolysis (PE) directly at the point of injury [13] and Percutaneous peripheral Nerve Stimulation (PNS) to stimulate the suprascapular nerve causing motor and sensory changes [14]. Both therapies are based on the application of direct current through needles, thanks to the ultrasound-guided development of the puncture [13]. This treatment causes an electrochemical reaction in the degenerated region of the tendon that facilitates collagen synthesis [15] and soft tissue regeneration [16,17,18], and reduces pain, and enhances movement, flexibility, strength and the development of tendon resistance over time [11,13].

Furthermore, updated interventions include Eccentric exercise (EE) to improve the resistance of non-contractile tissue thanks to the stimulation of fibroblasts, increasing collagen synthesis to reverse the pathological cycle [19,20,21,22].

The objective of this trial was to compare the effects of PE, PNS and EE with those of a well-established technique such as Transcutaneous Electrical Nerve Stimulation (TENS) and Therapeutic Ultrasound (US) for the management of supraspinatus tendinopathy [23,24,25]. The hypothesis of our work was that treatment by PE and PNS is better than TENS and US in relieving pain (NPRS); increasing strength; and improving the electromyographic activity, ultrasound characteristics of the tendon (echogenicity, thickness and hypervascularization) and functionality (DASH and SPADI) of patients with supraspinatus tendinopathy who simultaneously perform EE.

## 2. Materials and Methods

### 2.1. Study Design

This study is a randomized clinical trial with blinded evaluation by third parties, longitudinal and prospective. The research was developed following the Declaration of Helsinki and the CONSORT guidelines for clinical research. Approval was received from the Cádiz Research Ethics Committee (registration code 1681-N-21), and the protocol was registered at ClicialTrials.gov (NCT05627102). The intervention lasted 4 weeks; the study variables were evaluated pre-intervention and with follow-up in the post-treatment (4 weeks) and at 12 and 24 weeks.

### 2.2. Participants

The participants are individuals of both sexes between 18 and 65 years of age with painful symptoms associated with the supraspinatus tendon [10]: Shoulder pain in the insertion area of the supraspinatus tendon, which increases on palpation and is not associated with signs of root irritation; Structural changes in the tendon on ultrasound examination [26,27,28]; Reproducible pain in shoulder movements against resistance [10,29]; Positive result to Jobe, Neer and Hawkins–Kennedy tests [29].

Patients with any of the following conditions were excluded: bilateral involvement; previous treatment with PE or PNS in myotendinous pathology of the shoulder; recent severe trauma, surgery, previous shoulder fractures or dislocations; regular drug treatment; contraindicated invasive physiotherapy treatment (pregnant women, fibromyalgia, pacemaker patients, cancer patients, infectious processes or lymphedema).

All potential participants received the study information and signed the informed consent.

**Figure 1:**
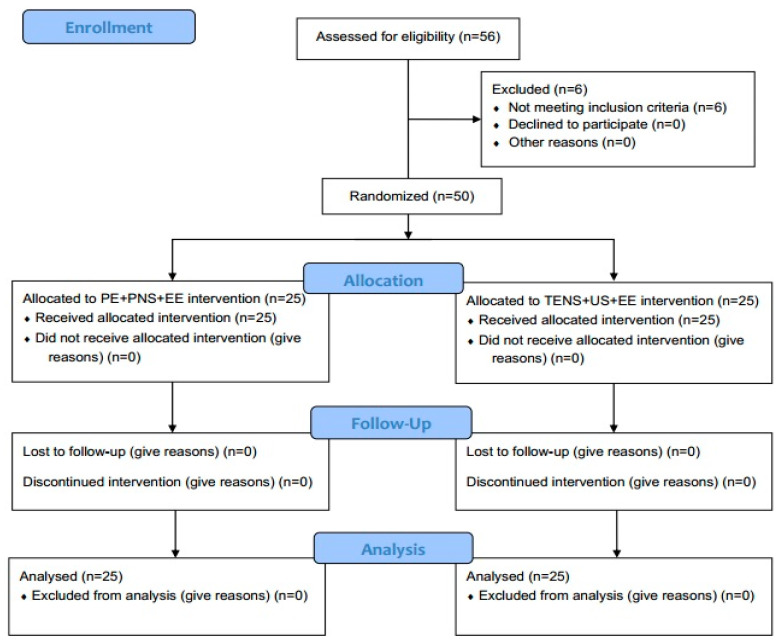
CONSORT flow diagram.

The CONSORT flow diagram of the sample is presented in Figure 1.

### 2.3. Sample Size Calculation, Randomization and Blinding

Epidat software version 3.1 (Servicio de Epidemiología de la Dirección Xeral de Saúde Pública da Consellería de Sanidade, Xunta de Galicia, Santiago de Compostela, Spain) is used. The calculation of the sample size is based on statistically significant differences of two units on the Numerical Pain Rate Scale (NPRS) [12], with a statistical power of 80% and setting a confidence level of 95%. Thus, it is determined that to carry out a study of these characteristics, the sample size must be at least of 18 individuals per group (36 in total); however, when carrying out long-term follow-up and in view of possible losses, the sample was expanded to 25 subjects per group (making a total of 50 participants).

Recruitment took place at the Policlínica Santa María Clinic (Cádiz, Spain). The participants were randomly divided into two equal groups with the AleatorMetod program, with an equal distribution (1:1). The initial assessment and follow-up measurements were made by a blinded evaluator without access to the distribution of the groups.

### 2.4. Outcomes Measurements

NPRS allows pain to be assessed through eleven levels, from 0 (absence of pain) to 10 (maximum pain). It is a validated instrument for pain assessment and shows excellent test–retest reliability [12,30,31].

Assessment of rotator cuff muscle strength may be related to the integrity of the rotator cuff [32,33,34]. Strength in abduction movements and internal and external rotations was assessed with a portable handheld dynamometer held by the evaluator [29].

Surface electromyography (EMG) involves the quantitative recording and graphic representation of the electrical signal produced in muscle contraction [35]. The electromyographic amplitude of the supraspinatus and upper trapezius is assessed during the maximum voluntary isometric contraction in the abduction movement against resistance [36]. The mDurance^®^ device (MDurance Solutions S.L., Granada, Spain) was used.

Ultrasound assessment analyzed changes in echogenicity, thickening and vascularization of the supraspinatus tendon [26,37]. The findings were compared with the unaffected contralateral shoulder. For each variable, the condition of positive or negative was registered. The ultrasound Mindray^®^ DP30 was used with a high-frequency linear probe, carrying out a transverse and longitudinal scan [26,27,28,37,38,39,40].

Disabilities of the Arm, Shoulder and Hand (DASH) is a specific tool for assessing the functionality and quality of life related to upper-limb pathologies [41]. The Shoulder Pain and Disability Index (SPADI) evaluates the patient’s perception regarding pain and disability associated with shoulder problems in their daily living activities [6,29,42].

### 2.5. Interventions

Two intervention groups were established, one with invasive physiotherapy treatment (PE+PNS) and the other with conventional electrotherapy treatment (TENS+US). Patients in both groups completed an EE program.

**Figure 2:**
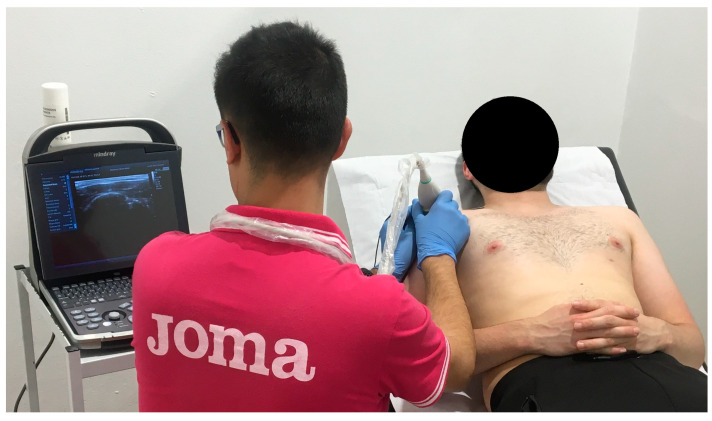
PE application in supraspinatus tendon.

PE application (Figure 2) was carried out once a week for four weeks. The intensity was 350 µA for 72 s [11,12]. The EPTE^®^ Bipolar System device (Ionclinics & Deionics S.L., Valencia, Spain) was used for treatment, and the Mindray^®^ DP30 ultrasound machine was used for Ultrasound-Guided localization of the target tissue [43]. The patient should be supine, with the shoulder in internal rotation, elbow semi-flexed and forearm pronated on his abdomen [11,12,43,44]. With the probe over the supraspinatus tendon, the needle (negative electrode) is inserted at an angle between 30° and 45°, with the axis of the tendon [11,44] and the surface positive electrode placed proximally over the upper trapezius muscle. Size 0.30 × 40 mm acupuncture needles are used [11,43,45].

**Figure 3:**
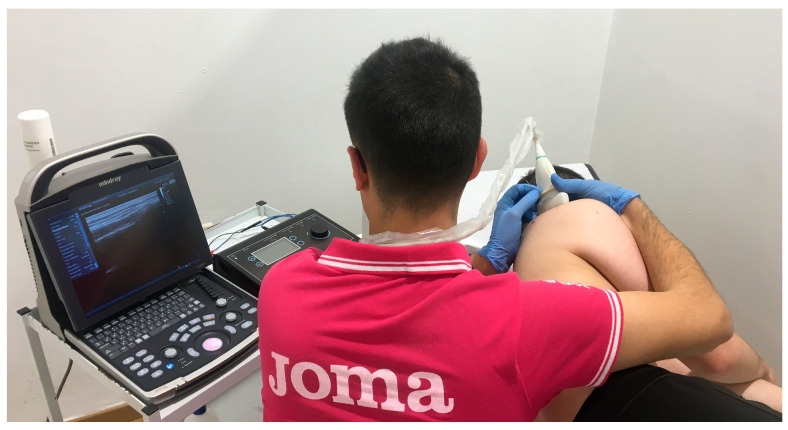
PNS application to suprascapular nerve.

PNS is carried out after the application of PE, with the same treatment frequency. The EPTE^®^ Bipolar System device (Ionclinics & Deionics S.L., Valencia, Spain) and the Mindray^®^ DP30 ultrasound machine were used. The needles are placed adjacent to the suprascapular nerve, deep to the upper trapezius and supraspinatus; the patient must be placed in lateral decubitus. A low-frequency current (10 Hz) is applied, seeking a sensory or motor response (at an intensity tolerable for the patient) for 90 s [14]. Size 0.30 × 40 mm acupuncture needles are used [43] (Figure 3).

Patients in the conventional electrotherapy treatment group received 10 sessions of TENS and US, five days per week. TENS was applied with the Megasonic 313 P4 device (Electromedicarin^®^, Barcelona, Spain) for 20 min, frequency of 150 Hz, pulse duration of 100 µs [46] and tolerable intensity [24,25]. The positive electrode was placed on the supraspinatus muscle and the negative electrode on the tendon [46] (conventional 5 × 9 cm electrodes were used). The Megasonic 212 K device (Electromedicarin^®^) was used for US treatment: 1 MHz frequency, 1.5 W/cm^2^ power, in continuous mode over the painful area for 5 min [25,47,48,49].

**Figure 4:**
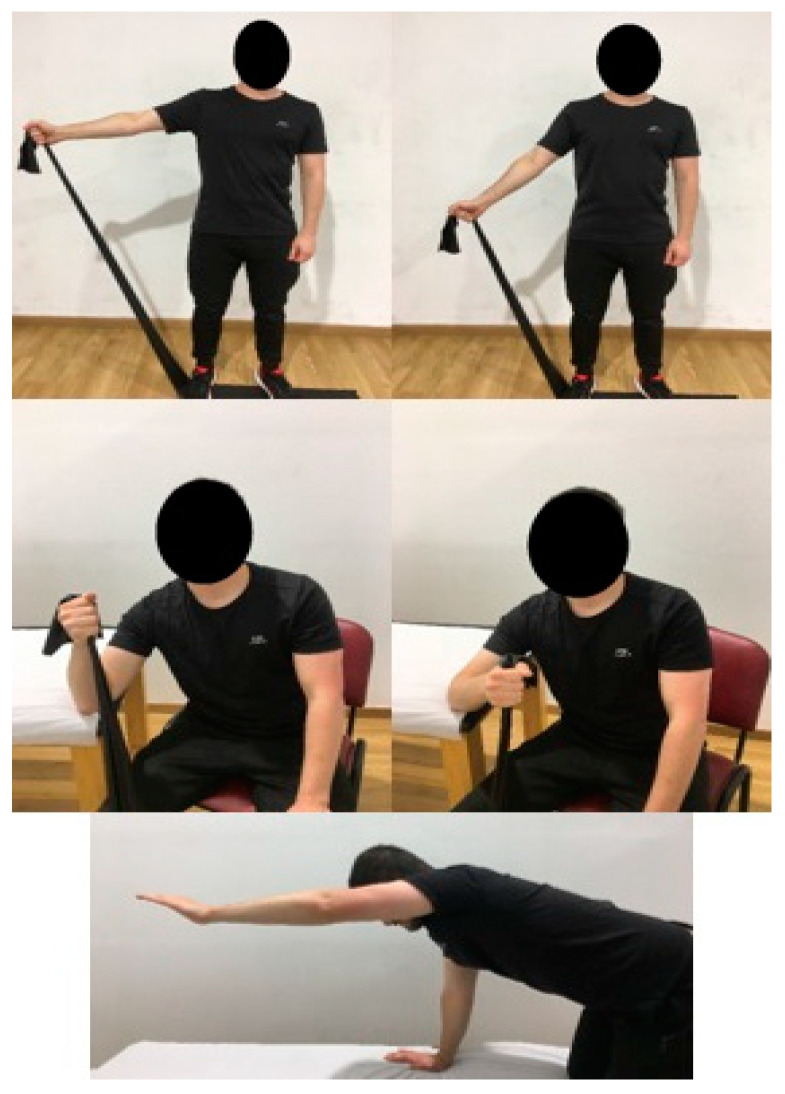
EE protocol.

The EE protocol includes 3 exercises, with 3 sets of 10 repetitions of each exercise, twice a day, for four weeks [11,19]. The first exercise focuses on the supraspinatus; the patient is standing and must perform shoulder abduction (concentric phase) followed by adduction of the same slowly (eccentric phase), with resistance from an elastic band [11,12,45]. The focus of the second exercise is the infraspinatus, from the seated position; the concentric phase will depend on external rotation, slowly returning to the initial position towards internal rotation [11,12,45]. The last of the exercises focuses on global shoulder stability; the subject is placed in a quadruped position, concentric phase in shoulder flexion and eccentric phase in the return movement [11,12,45] (Figure 4).

### 2.6. Statistical Analysis

The IBM SPSS Statistics package, version 23.0 (SPSS Inc., Chicago, IL, USA), was used for data management and analysis. The level of statistical significance was established at *p* < 0.05. Means and standard deviations were used to describe continuous variables, while categorical variables were described using frequencies and percentages. Levene’s test was used to analyze homoscedasticity, and the Kolmogorov–Smirnov test was used to analyze the normal distribution of continuous variables. Baseline comparability between groups was ensured using Student’s *t*-test for quantitative variables and the chi-square test (χ^2^) for categorical variables.

To assess the potential relationships between the ultrasound scan evaluation parameters and between the affected side and the dominant hand, Pearson’s correlation was used because of the bivariate nature of the variables.

To explore the differences between groups in ultrasound scan evaluations at each time point, a chi-square test was performed. This was due to the bivariate nature of the variables in the analysis, including group, hypoechogenicity, thickness and hypervascularity.

A 2 × 4 mixed model repeated measures analysis of variance (ANOVA) was used to analyze the main objective of this study. Time-by-group interaction was the correlation of interest. Variables with differences between the groups at baseline were analyzed by controlling for the effect of the variable at baseline. To test for differences between groups immediately after treatment, at 12 weeks of follow-up and at 24 weeks of follow-up, Student’s *t*-test was performed using pre- and post-change scores. Student’s *t*-test for paired samples was used to evaluate the differences within the groups between the two time points. The effect size (ES) for the time-by-group interaction of the 2 × 4 mixed ANOVA was assessed using eta-squared (η^2^). Additionally, Cohen’s d was used to assess the ES for the bivariate analysis. According to Cohen’s recommendations [50], η^2^ can be considered irrelevant when <0.02, small if between 0.02 and 0.15, medium if between 0.15 and 0.35 and large if >0.35. Similarly, a value of Cohen’s d < 0.2 can be considered irrelevant. Cohen’s d values were classified as small (0.2–0.49), medium (0.5–0.8) and large (>0.8) [50].

According to Mishra et al. [51], clinical success was defined as a 50% improvement in patient pain perception based on the NPRS scale. Furthermore, to determine the clinical significance of the study results, the number of need to treat (NNT) and the absolute risk reduction (ARR) were determined. NNT could be interpreted as the number of patients that needed to be treated with a therapy compared to another to achieve an additional beneficial result during a defined time lapse [52,53].

## 3. Results

A total of 50 patients completed all the tests and evaluations programmed in the study and were randomly assigned to 2 groups of 25 subjects (Figure 4). Men represented 72% of the sample, with a mean age of the total sample of 44.24 years old (SD = 11.80). During ultrasound exploration, it has been possible to observe hypo-echogenicity, thickness and hypervascularization signs in more than 86% of the sample, finding statistically significant correlations between echogenicity and thickness (r = 0.626; *p* < 0.001), echogenicity and hypervascularization (r = 0.626; *p* < 0.001), as well as between thickness and hypervascularization (r = 0.291; *p* = 0.040). Conversely, although patients presented a higher prevalence of the right hand as the dominant hand, as well as a greater prevalence of the right hand as the affected arm, the analysis has not revealed statistically significant correlations between the dominant hand and the affected arm (r = 0.007; *p* = 0.960). All morphologic and baseline data are presented in Table 1.

The results of the analysis of the variance performed to evaluate the effect of the experimental therapy through time showed statistically significant differences for all study variables with effect sizes between medium and large and power values between 0.777 and 1.000 (Table 2).

At immediate post-treatment, although the paired sample *t*-test to evaluate within-groups differences revealed statistically significant improvements in both groups for all study variables (Table 3), the between-groups analysis showed greater statistically significant enhancements in favor of the PE+PNS+EE group for all study variables (Table 3). Furthermore, all significant variables showed large effect size values ranged between 0.879 and 2.117, except for internal rotation strength (d = 0.691) and trapezius muscular recruitment (d = 0.710) that showed medium effect size values (Table 3). Concerning the established clinical success criteria, the PE+PNS+EE group showed a clinical success of 80% (20 patients), while the TENS+US+EE group showed a clinical success of 32% (8 patients) at immediate post-treatment. Thus, in the case of the PE+PNS+EE treatment, the probability of success increases by 48% (ARR = 0.48; 95%IC = 0.24 to 0.72). In other words, 2.083 subjects are needed to obtain at least one favorable outcome compared to the TENS+US+EE group (NNT = 2.083; 95%IC = 1.39 to 4.18) at immediate post-treatment. Moreover, at this time-point evaluation in the ultrasound scan evaluation, statistically significant differences have been only observed in hypervascularization (Echogenicity χ^2^ = 1.495; *p* = 0.221/Thickness χ^2^ = 0.355; *p* = 0.552/Hypervascularization χ^2^ = 9.921; *p* = 0.002), being able to observe a reduction of positive cases in the PE+PNS+EE group ranged between 8 and 40 percentage points in the ultrasound scan evaluation (Table 4).

At the 12-weeks follow-up, the within-groups analysis also revealed statistically significant improvements in both groups for all study variables (Table 3), but in the same way as immediate post-treatment analysis, the between-groups analysis showed greater statistically significant enhancements (Table 3). Additionally, all significant variables showed large effect size values ranged between −0.883 and −2.358. Concerning the established clinical success criteria, the experimental group showed a clinical success of 92% (23 subjects); meanwhile, the control group showed a clinical success of 68% (17 subjects) at the three-months follow-up. Therefore, in the case of the experimental treatment, the probability of success increases by 24% (ARR = 0.24; 95%IC = 0.03 to 0.45). In other words, 4.167 persons are needed to obtain at least one favorable outcome compared to the control group (NNT = 4.167; 95%IC = 2.21 to 35.13) at the three-months follow-up. In addition, it is in favor of the PE+PNS+EE group for all study variables (Table 4). In addition, at this time-point evaluation, statistically significant differences were appreciated between the PE+PNS+EE and TENS+US+EE groups percentages for all ultrasound scan evaluations (Echogenicity χ^2^ = 3.947; *p* = 0.047/Thickness χ^2^ = 12.000; *p* = 0.001/Hypervascularization χ^2^ = 11.688; *p* = 0.001), being able to observe a reduction of positive cases in the PE+PNS+EE group ranged between 24 and 60 percentage points in the ultrasound scan evaluation (Table 4).

At the 24-weeks follow-up, the within-groups analysis revealed statistically significant improvements in both groups for all study variables (Table 3), but in the same way as at immediate post-treatment and at the 12-weeks follow-up, the between-groups analysis showed greater statistically significant enhancements in favor of the PE+PNS+EE group for all study variables (Table 3). Concerning the established clinical success criteria, the experimental group showed a clinical success of 92% (23 subjects); meanwhile, the control group showed a clinical success of 72% (18 subjects) at the six-months follow-up. Therefore, in the case of the experimental treatment, the probability of success increases by 20% (ARR = 0.20; 95%IC = −0.01 to 0.41). In other words, five persons are needed to obtain at least one favorable outcome compared to the control group (NNT = 5; 95%IC = 2.47 to −177.26) at the six-months follow-up. In orthopedic tests (Neer test X^2^ = 6.640; *p* = 0.010/Hawking Kennedy test X = 10.784; *p* = 0.001/Jobe test X^2^ = 3.309; *p* = 0.069) and for all ultrasound scan evaluations (Echogenity X^2^ = 8.117; *p* = 0.004/Thickness X^2^ = 14.346; *p* < 0.001/Vascularity X^2^ = 13.235; *p* < 0.001), being able to observe a reduction of positive cases in the experimental group ranged between 80 and 96 percentage points in the orthopedic tests and between 52 and 84 percentage points in the ultrasound scan evaluation (Table 4).

## 4. Discussion

The effects achieved with the treatment of PE+PNS+EE offer statistically significant improvements superior to the treatment protocol of TENS+US+EE on variables analyzed, both in the direct post-intervention assessment and in the follow-up measurements.

The combination of PE, PNS and EE has superior clinical success (pain reduction and structural changes), the effect occurs in the shorter term (at the end of the treatment) and is maintained over time. Therefore, it would be possible to consider that these treatments favor the repair of the injured structure and control the nociceptive stimulus of local origin, causing an increase in functionality [54,55,56].

PE produces a localized inflammatory response on the injured tendon, thus initiating the repair mechanisms [57], thanks to the mechanical stimulus, the modification of the pH [58] and the activation of the NLRP3 inflammasome that promotes collagen synthesis [59,60] and matrix remodeling [61]. In addition, PNS causes stimulation of Aβ nerve fibers, which blocks nociceptive information from the Aδ and C fibers [14,62]. This may be linked to the control of pain perception and also to the optimization of muscle function [14,62,63], taking into account the relationship between the suprascapular nerve and rotator cuff tendinopathy [64,65,66].

On the other hand, exercise makes it possible to achieve changes in pain perception [56] and provides mechanical loading stimulus; this enables tendon development and longitudinal collagen alignment [67], and increases the cross section, muscle strengthening and greater resistance capacity of the tendon [19,68]. The EE program represents the common point between the treatments, and intragroup improvements were found in both groups at follow-up. The changes in the TENS+US+EE group appear in the longer term and could be related to the effects derived from the EE.

In both treatment groups, higher strength levels were achieved in the post-intervention follow-up evaluations compared to baseline strength. These changes are statistically significant and attributable to the EE in the protocols. Loss of strength could be a predisposing factor for shoulder injuries [69]. Through EE, muscle strengthening is achieved, and the capacity of the tendon increases [70].

Along the same lines, the increase in strength levels is also reflected in electromyographic activity. In the analysis of the basal condition, it is observed that in the subjects of both groups there are average levels of activation of the upper trapezius above the supraspinatus in the abduction movement, taking into account that pain and dysfunction modify glenohumeral and scapulothoracic neuromuscular recruitment patterns [35].

The intragroup analysis indicates that in both groups, there is progress towards greater activation of the supraspinatus and a stabilization of the signal amplitude of the upper trapezius. With PE+PNS+EE treatment, there is a greater increase in the electromyographic activity of the supraspinatus. This could indicate that better biomechanical performance is achieved when the treatment produces analgesia and reversal of the pathological cycle.

In the ultrasound evaluation, focal hypoechoic areas [37], thickening as part of the degenerative process [37] (there is no classic prostaglandin inflammation [71]), neovascularization and neoinnervation as a result of a failed repair process [37] are observed. There is a statistically significant correlation between echogenicity and thickening, echogenicity and vascularization, and thickening and vascularization.

In the post-intervention results, a greater reduction in positive cases was found in the PE+PNS+US group than in the TENS+US+EE group. Therefore, the combination of PE+PNS+US could be associated with greater structural changes in the tissue and in symptomatology.

DASH [41] and SPADI [6,42] questionnaires express the degree of disability of the patient, which could be explained as the result of the combination of painful perception and limitation of movement. In the intragroup analysis of the pre- and post-intervention differences, changes appear in both treatment groups.

However, the comparison between groups offers statistically significant differences favorable to the PE+PNS+EE group, the same results as in research that includes PE+EE [11,45] or different types of exercise [72].

The strong point of this study is the inclusion of two invasive physiotherapy techniques at the forefront in the clinical field; long-term monitoring of study variables; and the inclusion of ultrasound analysis, electromyographic activity of the supraspinatus muscle and muscle strength in rotator cuff movements. Invasive techniques are safe and innovative, and exercise encourages the active role of the patient, which could also improve their physical condition and promote a healthier lifestyle.

There are limitations because the invasive physiotherapy treatments applied depend on the physiotherapist, and performing EE daily depends on the patient. Likewise, the ultrasound study of the tendon has been evaluated qualitatively and is dependent on the evaluator. Also, in evaluations dependent on the sensation of the individual, patients must remember their initial state and compare it. In addition, different pathological stages could occur with the same diagnosis, which could condition the results of the treatments. And the therapy could have a relatively high cost and depends on the training of the physiotherapist.

A more in-depth analysis of EMG could be included in future studies: contralateral comparison; assessment of more muscle groups and in different movements or types of contraction; evaluation of the muscle activation sequence; and the assessment combined with other variables (electromyographic activity and dynamometry). Equally, in future research it would be advisable to separately analyze the effects of each proposed intervention, to include greater blinding conditions (sham invasive treatment) and different dosages of the treatments, and to analyze the effects on tendons in other areas of the body or incorporate a control group without treatment. The future is to continue researching to improve the care of each patient, adapting the treatment individually to their pathology. It is important to accompany pain education treatments, avoiding catastrophism and fragility of the patient. This type of research aims to provide answers to patients and professionals to guarantee effective action protocols.

## 5. Conclusions

The combined treatment of PE, PNS and EE is an effective option in the clinical management of tendinopathies, with positive results in the short and long term. Statistically significant improvements appear in pain (NPRS), strength in abduction movements and internal and external rotations, electromyographic activity of the supraspinatus in shoulder abduction, ultrasound characteristics of the tendon (thickening, echogenicity and hypervascularization) and functionality shoulder (DASH and SPADI) with the intervention parameters proposed in this study. The results obtained are especially relevant due to their translation to clinical practice. Therefore, the treatment may be recommended for patients with supraspinatus tendinopathy.

## Figures and Tables

**Table 1:** Morphological and clinical characteristics of the sample and between-group comparison at baseline.

		All (50)		PE+PNS+EE (16)		TENS+US+EE (16)		
Categorical		Frequency	%	Frequency	%	Frequency	%	*p*
Sex	Male	36	72.0	19	76.0	17	68.0	0.529
	Female	14	28.0	6	24.0	8	32.0	
Hypoechogenicity	Yes	43	86.0	22	88.0	21	84.0	0.684
	No	7	14.0	3	12.0	4	16.0	
Thickness	Yes	47	94.0	24	96.0	23	92.0	0.552
	No	3	6.0	1	4.0	2	8.0	
Hypervascularization	Yes	47	94.0	23	92.0	24	96.0	0.552
	No	3	6.0	2	8.0	1	4.0	
Affected side	Right	34	68.0	16	64.0	18	72.0	0.544
	Left	16	32.0	9	36.0	7	28.0	
Dominant hand	Right	47	94.0	24	96.0	23	92.0	0.552
	Left	3	6.0	1	4.0	2	8.0	
**Continuous**		**Mean**	**SD**	**Mean**	**SD**	**Mean**	**SD**	***p***
Age		44.24	11.80	44.36	13.36	44.12	10.29	0.944
Weight		82.08	11.44	83.88	7.50	80.28	14.28	0.270
Height		1.74	0.07	1.74	0.07	1.74	0.07	0.752
BMI		27.17	4.10	27.66	2.81	26.68	5.09	0.402
NPRS		7.24	1.36	7.56	1.26	6.92	1.41	0.097
DASH		54.50	19.40	62.16	15.86	46.84	19.86	0.004
% DASH		45.42	16.16	51.80	13.22	39.03	16.55	0.004
SPADI		69.32	18.75	71.24	17.50	67.40	20.09	0.475
% SPADI		53.32	14.42	54.80	13.46	51.85	15.46	0.475
Abduction strength		8.45	1.42	8.56	1.74	8.34	1.03	0.590
Internal rotation strength		8.55	1.42	8.70	1.68	8.41	1.12	0.480
External rotation strength		8.40	1.39	8.42	1.54	8.38	1.26	0.920
Supraspinatus EMS		168.31	89.32	162.73	104.37	173.88	73.01	0.663
Upper trapezius EMS		391.98	165.12	400.94	173.91	383.02	158.92	0.705

Abbreviatures. %: Percentage; *p*: *p*-value; BMI: Body Mass Index; DASH: Disabilities of the Arm, Shoulder and Hand; EE: Eccentric exercise; EMS: Surface Electromyography; NPRS: Numerical Pain Rate Scale; PE: Percutaneous Electrolysis; PNS: Percutaneous peripheral Nerve Stimulation; SD: Standard Deviation; SPADI: Shoulder Pain and Disability Index; TENS: Transcutaneous Electrical Nerve Stimulation; US: Therapeutic Ultrasound.

**Table 2:** Statistical significance, effect size and power of time-by-group interaction from 2 × 4 mixed model repeated measures ANOVA.

Variable	F	*p*	η^2^	Power
NPRS	17.684 **	0.000	0.536	1.000
DASH	22.962 **	0.000	0.600	1.000
% DASH	22.962 **	0.000	0.600	1.000
SPADI	21.328 **	0.000	0.582	1.000
% SPADI	21.328 **	0.000	0.582	1.000
Abduction strength	6.086 *	0.001	0.284	0.945
Internal rotation strength	3.754 *	0.017	0.197	0.777
External rotation strength	8.340 **	0.000	0.352	0.988
Supraspinatus EMS	17.443 **	0.000	0.532	1.000
Upper trapezius EMS	5.703 *	0.002	0.271	0.929

Abbreviatures. η^2^: Eta-squared; *p*: *p*-value; DASH: Disabilities of the Arm, Shoulder and Hand; EMS: Surface Electromyography; SPADI: Shoulder Pain and Disability Index. *: *p* < 0.05; **: *p* < 0.001.

**Table 3:** Paired sample *t*-test and Student’s *t*-test for within-groups and between-groups differences in post-treatment, 12-weeks follow-up and 24-weeks follow-up.

Variable		Post-Treatment					12-Weeks Follow-Up					24-Weeks Follow-Up				
		Within-Group Change Score		Between-Groups Change Score		Effect Size	Within-Group Change Score		Between-Groups Change Score		Effect Size	Within-Group Change Score		Between-Groups Change Score		Effect Size
		Mean Dif.	*p*	Mean Dif.	*p*	d	Mean Dif.	*p*	Mean Dif.	*p*	d	Mean Dif.	*p*	Mean Dif.	*p*	d
NPRS	PE+PNS+EE	−5.48	0.000 **	−2.96	0.000 **	1.527	−6.22	0.000 **	−2.74	0.000 **	−1.39	−6.60	0.000 **	−3.08	0.000 **	−1.857
	TENS+US+EE	−2.52	0.000 **				−3.48	0.000 **				−3.52	0.000 **			
DASH	PE+PNS+EE	−48.16	0.000 **	−33.16	0.000 **	2.117	−55.12	0.000 **	−36.56	0.000 **	−2.358	−57.20	0.000 **	−37.44	0.000 **	−2.374
	TENS+US+EE	−15.00	0.000 **				−18.56	0.000 **				−19.76	0.000 **			
% DASH	PE+PNS+EE	−40.13	0.000 **	−27.63	0.000 **	2.116	−45.93	0.000 **	−30.47	0.000 **	−2.357	−47.67	0.000 **	−31.20	0.000 **	−2.348
	TENS+US+EE	−12.50	0.000 **				−15.47	0.000 **				−16.47	0.000 **			
SPADI	PE+PNS+EE	−52.84	0.000 **	−30.96	0.000 **	2.016	−61.04	0.000 **	−33.64	0.000 **	−2.02	−63.96	0.000 **	−35.36	0.000 **	−2.133
	TENS+US+EE	−21.88	0.000 **				−27.40	0.000 **				−28.60	0.000 **			
% SPADI	PE+PNS+EE	40.65	0.000 **	−23.82	0.000 **	2.016	−46.95	0.000 **	−25.88	0.000 **	−2.02	−49.20	0.000 **	−27.20	0.000 **	−2.132
	TENS+US+EE	16.83	0.000 **				−21.08	0.000 **				−22.00	0.000 **			
Abduction strength	PE+PNS+EE	1.42	0.000 **	0.74	0.000 **	1.132	1.81	0.000 **	0.83	0.000 **	1.169	2.18	0.000 **	1.10	0.000 **	1.178
	TENS+US+EE	0.68	0.000 **				0.98	0.000 **				1.09	0.000 **			
Internal rotation strength	PE+PNS+EE	1.10	0.000 **	0.32	0.030 *	0.637	1.60	0.000 **	0.56	0.002 **	0.911	1.82	0.000 **	0.66	0.004**	0.868
	TENS+US+EE	0.78	0.000 **				1.04	0.000 **				1.16	0.000 **			
External rotation strength	PE+PNS+EE	1.40	0.000 **	0.58	0.000 **	1.065	1.87	0.000 **	0.81	0.000 **	1.193	2.22	0.000 **	1.02	0.000 **	1.408
	TENS+US+EE	0.82	0.000 **				1.06	0.000 **				1.20	0.000 **			
Supraspinatus EMS	PE+PNS+EE	85.36	0.000 **	67.82	0.000 **	1.890	127.57	0.000 **	96.16	0.000 **	1.958	155.67	0.000 **	122.21	0.000 **	1.881
	TENS+US+EE	17.54	0.000 **				31.40	0.000 **				33.46	0.000 **			
Upper trapezius EMS	PE+PNS+EE	−136.96	0.000 **	−71.05	0.016 *	0.710	−167.15	0.000 **	−95.92	0.003 **	−0.883	155.67	0.000 **	−78.50	0.015 *	−1.719
	TENS+US+EE	−65.91	0.000 **				−71.23	0.000 **				−88.81	0.000 **			

Abbreviatures. d: Cohen’s d; *p*: *p*-value; DASH: Disabilities of the Arm, Shoulder and Hand; EE: Eccentric exercise; EMS: Surface Electromyography; Mean dif.: Mean difference; NPRS: Numerical Pain Rate Scale; PE: Percutaneous Electrolysis; PNS: Percutaneous peripheral Nerve Stimulation; SPADI: Shoulder Pain and Disability Index; TENS: Transcutaneous Electrical Nerve Stimulation; US: Therapeutic Ultrasound. *: *p*-value < 0.05; **: *p*-value < 0.01.

**Table 4:** Frequencies and percentages of categorical variables at post-treatment, 12-weeks follow-up and 24-weeks follow-up.

Variable			Post-Treatment		12-Weeks Follow-Up		24-Weeks Follow-Up	
			Frequency	%	Frequency	%	Frequency	%
Hypoechogenicity	PE+PNS +EE	Yes	20	80.0	16	64.0	9	36.0
		No	5	20.0	9	36.0	16	64.0
	TENS+US+EE	Yes	23	92.0	22	88.0	19	76.0
		No	2	8.0	3	12.0	6	24.0
Thickness	PE+PNS +EE	Yes	19	76.0	9	36.0	3	12.0
		No	6	24.0	16	64.0	22	88.0
	TENS+US+EE	No	23	92.0	21	84.0	16	64.0
		No	2	8.0	4	16.0	9	36.0
Hypervascularization	PE+PNS +EE	Yes	13	52.0	8	32.0	2	8.0
		No	12	48.0	17	68.0	23	92.0
	TENS+US+EE	Yes	23	92.0	20	80.0	14	56.0
		No	2	8.0	5	20.0	11	44.0

Abbreviatures. %: Percentage; EE: Eccentric exercise; PE: Percutaneous Electrolysis; PNS: Percutaneous peripheral Nerve Stimulation; TENS: Transcutaneous Electrical Nerve Stimulation; US: Therapeutic Ultrasound.

## Data Availability

Data are contained within the article.
